# Optical, Morphology, and Structural Characterization of Synthesized Eco‐Friendly Silver Nanoparticles for Potential Antibacterial Applications

**DOI:** 10.1155/tswj/7155762

**Published:** 2026-10-28

**Authors:** Hatem Abdullah Al-Ameryeen, Mou′ad. A. Tarawneh, Hakim Al Garalleh, Lih Jiun Yu

**Affiliations:** ^1^ Department of Physics College of Science Al-Hussein Bin Talal University Ma′an Jordan ahu.edu.jo; ^2^ Department of Mathematical Sciences College of Engineering University of Business and Technology Jeddah Saudi Arabia ubt.edu.sa; ^3^ Faculty of Engineering Technology and Built Environment UCSI University Kuala Lumpur Malaysia ucsiuniversity.edu.my; ^4^ UCSI-Cheras Low Carbon Innovation Hub Research Consortium Kuala Lumpur Malaysia

**Keywords:** biosynthesis, eco-friendly, environmental science, green product, optical properties, plant extract, silver nanoparticles

## Abstract

In this work, the silver nanoparticles (AgNPs) were synthesized by an eco‐friendly biosynthesis using *Bougainvillea spectabilis* leaf extract, which acts as reducing and capping agent. The AgNPs were analyzed using UV spectroscopy, FTIR spectroscopy, XRD, and scanning electron SEM. From the UV‐visible spectral analysis we recorded an SPR peak at 415 nm, which is characteristic of spherical AgNPs. The results obtained from FTIR suggest the potential role of biomolecules in reducing Ag^+^ and the capping of AgNPs. The XRD pattern demonstrated the FCC crystalline of metallic silver, showing average crystallite size ranging from 30 to 50 nm. Results obtained from the SEM revealed the morphology and size distribution of synthesized AgNPs. From the images obtained, we determined a spherical morphology with an estimated size distribution measuring 50–200 nm. Furthermore, the AgNPs exhibited strong antibacterial behavior against tested bacterial strains, namely *Staphylococcus aureus* and *Pseudomonas aeruginosa*. These results highlight that the silver NPs are sustainable for potential biomedical and environmental applications.

## 1. Introduction

The scientific field has demonstrated much interest in the application of nanomaterials, attributed to their unique properties. The application areas include biomedical fields, environment, and others [1]. The promising areas in the application of nanomaterials include biomedicine, drug delivery, biolabeling, bioimaging, photocatalysis, photovoltaic, and solar cells [2]. Silver nanoparticles (AgNPs) gained significant concentration because of the diminishing efficacy of conventional antibiotics and rapid emergence of highly resistant microbes [3]. There are multiple routes available in the synthesis of AgNPs. The rapidly growing branch of nanotechnology has made significant progress in the preparation and usage of AgNPs because of their extraordinary antibacterial properties, thanks to their distinctive optical, morphological, and structural properties. These nanoparticles have high surface volume ratio, LSPR, and ROS production properties, which work in combination to enhance their potency against bacterial organisms. However, the agents employed in preparing AgNPs are toxic, costly, and result in the production of eco‐toxic compounds.

Recently, biological methods have provided an advantageous, nontoxic alternative to the conventional chemical method used for producing AgNPs [4]. The green synthesis technique offers advantages over other chemical and physical methods, wherein the process is more economical, eco‐friendly, and easily adaptable on a large scale without the need to apply energy, high pressure, high temperatures, or toxic chemicals [5]. There has been an increasing demand in the emerging economical, eco‐friendly, and green way of synthesizing nontoxic chemicals in preparing AgNPs. Green alternative synthesis of AgNPs by microbes, plants, and algae offers an obvious natural, biologically safe, and environmentally friendly technique. Plants might be much more advantageous in AgNP synthesis compared with the use of bacteria and other chemicals since there is almost zero possibility of bacterial and dangerous chemical contaminants with significantly reduced usage of energy with vast implications and applications [6]. There has been significant progress in the past few years in terms of the biological synthesis of AgNPs using extracts of plants and microbes acting as reducing agents, along with their antimicrobial properties. The biomolecules flavonoids, carboxylic acids, aldehydes ketones, phenols, tannins, and proteins found in plant extracts were found to be accountable agents in reducing silver ions (Ag^+^) to metallic silver (Ag^0^), synthesizing AgNPs. The shape, size, morphology, and sizes of the preparation of AgNPs differed. These factors were strongly affected by experimental parameters, such as temperatures, pH, kinetic interactions, kind, and capping efficiency of plant biomolecules. The AgNPs properties are largely reliant on their shape, size, morphology, and size distribution [4, 7]. Consequently, the design controlling size, shapes, stabilizing, and altering the properties in nanoparticles was found to be the most significant part of the overall nanoparticle source in current related scientific investigations utilizing nanoparticle compounds [8, 9].

Although the mechanism of nanoparticle formation by several plant extracts is unclear, one possible explanation is that biomolecules in the plant extracts, for example proteins, phenols, and flavonoids play a critical role in reducing metal ions in addition to capping the formed nanoparticles [10]. Consistent with previous literature, the bioreduction process occurs in three major steps, including (1) reduction and nucleation of silver ions, (2) the aggregation stage and growth, and (3) stabilization in the terminal stage and capping. Phytochemicals found in plants play important roles, including secondary compounds such as sugars, polyphenols, ketones, phenolic acids, proteins, amides, and terpenoids. Likewise, in most cases, the reducing agent in the plant extract also functions simultaneously as a stabilizing agent and capping [11].

AgNPs show pronounced antibacterial activities and inhibition properties and have been used for preventing and treating diseases [12–16]. Lately, significant research has focused on identifying effective, nontoxic, and natural compounds with high antioxidative activities. The high antioxidant property shown by some nanomaterials opens promising avenues in designing innovative treatment approaches with higher and target‐specific actions. For example, silver, gold, and selenium nanoparticles have been demonstrated to have the potency to decrease the levels of oxidative stress because of their high redox‐active radical‐scavenging plants with antimicrobial properties have been found to be of much interest in terms of utilizing them in the eco‐friendly preparation of AgNPs to effectively treat microbial diseases due to the intricacy of their phytochemicals [16–20]. However, investigating plants in the preparation of AgNP is also an important topic of current interest [16, 20]. Understanding the chemical characterization of green‐synthesized AgNPs helps in establishing the correlation between their optical properties investigated by utilizing UV‐Vis spectroscopy in terms of LSPR peaks, sizes, and distributions [21, 22]. Furthermore, morphological and structural characterization techniques such as TEM, scanning electron microscopy (SEM), x‐ray diffraction (XRD), and FTIR, respectively, display complex information describing the shapes, sizes, and functionalization properties of nanoparticles [14, 15]. The bougainvillea plants are broadly recognized and traditionally valued for their colorful bracts in tropical regions; they also contain various bioactive compounds functioning as stabilizing and reducing agents, making them highly suitable for advanced nanotechnology and biomedical applications.

Consequently, these biogenic components prevent agglomeration and enhance structural stability. To explore this green advantage, this study investigated the physicochemical characterization of synthesized nanoparticles using *Bougainvillea spectabilis* leaf extract, including their structural, optical, and morphological analyses. In addition, their antibacterial effectiveness against selected bacterial strains was evaluated.

## 2. Materials and Methods

### 2.1. Materials

Leaves of *B*. *spectabilis* were collected from the botanical gardens of Al‐Hussein Bin Talal University in Maan, Jordan. The collected leaves were first prewashed with water to remove dust particles and later rinsed with distilled water. The clean leaves were dried and ground into powder using a grinder. Silver nitrate (AgNO_3_) (99.8% purity) was obtained from Sigma‐Aldrich.

### 2.2. Material Preparation

#### 2.2.1. Leaf Extracts

For preparing the extract, 50 g of finely powdered *B*. *spectabilis* leaves were mixed with 400 mL of deionized water. The mixture was heated to a temperature of 100°C for a period of 30 min, followed by the filtration of the extract obtained with the help of 90‐mm filter paper. The resulting filtrate was stored for the biosynthesis of AgNPs at 4°C.

##### 2.2.1.1. AgNPs Synthesis

A green synthesis technique was employed to develop AgNPs. The AgNO_3_ used as a precursor in the preparation of AgNPs from *B*. *spectabilis* leaf extract. A concentration of 1‐mM AgNO_3_ is prepared in double‐distilled water, which is refrigerated at a temperature of 4°C. To induce the reduction of Ag + ions, 1 mL of bougainvillea leaf extract was slowly added to 100 mL of an aqueous 1 mM of AgNO_3_ solution at a controlled initial pH of approximately 6.8. The mixture was then heated to a constant temperature of 75°C under constant magnetic stirring at 500 rpm for 1 h. The reaction mixture centrifuged at 5000 rpm for 30 min, then the resulting pellet washed five times with deionized water and once with absolute ethanol to remove uncoordinated biomolecules. Hence, the obtained AgNP yields were refrigerated at 4°C in the dark to ensure that the sample was not in a state of agglomeration. A color change indicated the successful production of AgNPs. The plant sample was collected and prepared, as well as a change in color after adding AgNO_3_, is indicated in Figure 1. A graphic process of the green synthesis of AgNPs is demonstrated in Figure 2.

**Figure 1:**
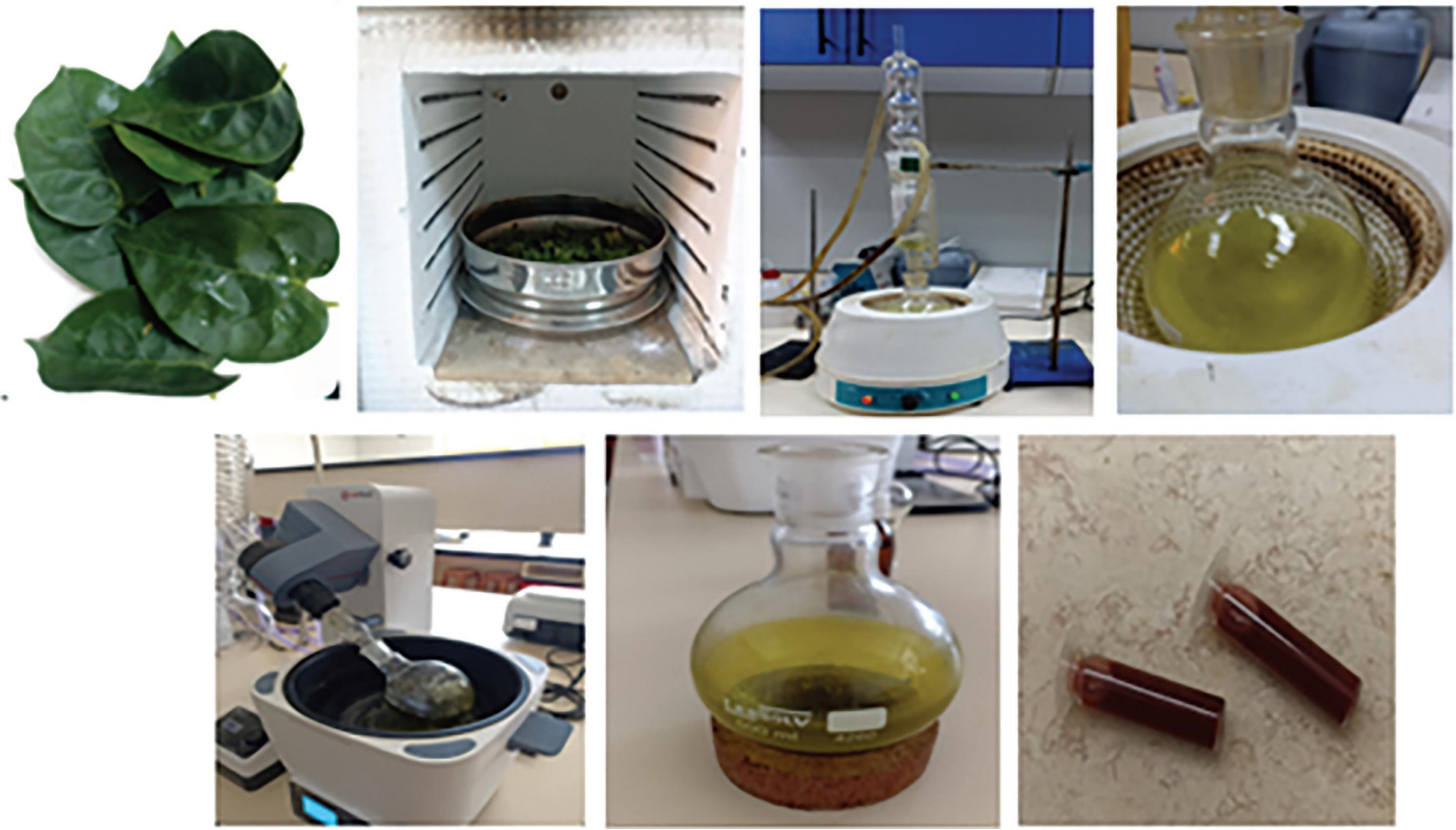
The green synthesis steps for silver nanoparticles.

**Figure 2:**
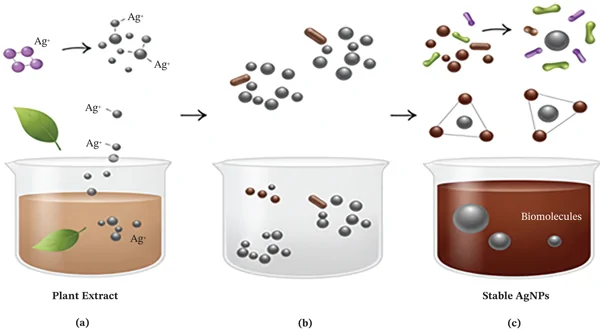
The mechanism of green synthesis of silver nanoparticles. (a) Reduction and nucleation. (b) Growth and aggregation. (c) Capping and stabilization.

#### 2.2.2. Characterizations

A UV‐Vis spectrometer (Shimadzu, UV‐1800) operating within wavelength range from 200 to 1000 nm with a high‐resolution 1‐nm bandwidth was used to identify the maximum surface plasmon resonance (SPR), which is revealed by an absorbance peak, characteristic of the formation of AgNPs.

FTIR analyses were performed using an FTIR spectrometer (PerkinElmer Spectrum LS‐65 luminescence, United States). The FTIR spectra of the synthesized AgNPs ranged from 4000 to 400 cm^−1^. The analysis was performed to identify the organic functional groups shown on the surface of the AgNPs that act as primary reduction and stabilization/capping agents.

An XRD study of the AgNPs was examined by a Shimadzu XRD‐6000 device (Shimadzu Corporation, Kyoto, Japan) within angles scanning of 10°–80°. The resulting data was used to consider the crystalline phase and verify the average crystallite size of the nanoparticles using the Debye–Scherrer equation (Equation (1)):

(1) D=k.λβ.cosƟ,

where *D* is the average particle size (nm), *K* denotes the shape factor (0.9), *λ* is the wavelength (0.15406 nm for Cu‐K*α*), *β* is the FWHM, and *θ* is the Bragg diffraction angle (degree).

The surface morphology of the AgNPs was observed by a SEM (FEI Nova NanoSEM 450, United States). A droplet of samples was left on a carbon‐coated copper grid until completely dry prior to imaging.

#### 2.2.3. Evaluation of Antimicrobial Activity

The antibacterial efficacy of the AgNPs was assessed against *Staphylococcus aureus* (gram‐positive, Reference ATCC 25923) and *Pseudomonas aeruginosa* (gram‐negative, Reference ATCC 27853) strains, obtained from Microbiology Research Laboratory, Department of Biology at AHU. The microbroth dilution method was used to find the minimum inhibitory concentrations (MIC) across three independent synthesis batches. The evaluation included three independent batches evaluated across three distinct experimental runs, with each analyzed in technical triplicate (*n* = 3). Two‐fold serial dilutions were established within Mueller–Hinton Broth. AgNPs and leaf extracts were tested in a concentration range of 1000–1.95 *μ*g/mL, whereas AgNO_3_ was evaluated at 500–0.98 *μ*g/mL. Bacterial cultures (24‐h growth) were standardized to 0.5 McFarland (1.5 × 10^8^ cells/mL) using 0.9% saline. An inoculum of 10^6^ cells/mL was combined with equivalent volumes of test materials and appropriate controls (growth and sterility) and then kept at 37°C for 24 h. Visual turbidity indicates bacterial growth, whereas clearly well signified growth inhibition. The MIC was established as the lowest concentration exhibiting no obvious growth, yielding identical modal MIC values across all experimental replicates to get high statistical reproducibility and reliability across all independent runs.

## 3. Results and Discussion

### 3.1. UV Spectroscopy Analysis

The green synthesis of AgNPs was initially determined by a notable color transition of the reaction mixtures from yellowish green to brown. This phenomenon of color change is directly related to the SPR of free conduction electrons on the surface of the AgNPs.

The UV‐Vis results as shown in Figure 3, verified that synthesized AgNPs exhibit a sharp and well‐defined SPR peak at 415 nm (with a corresponding maximum absorption at 404 nm). In accordance with previous literature, this absorption signature corresponds to spherical AgNPs with dimensions ranging between 40 and 100 nm [22].

**Figure 3:**
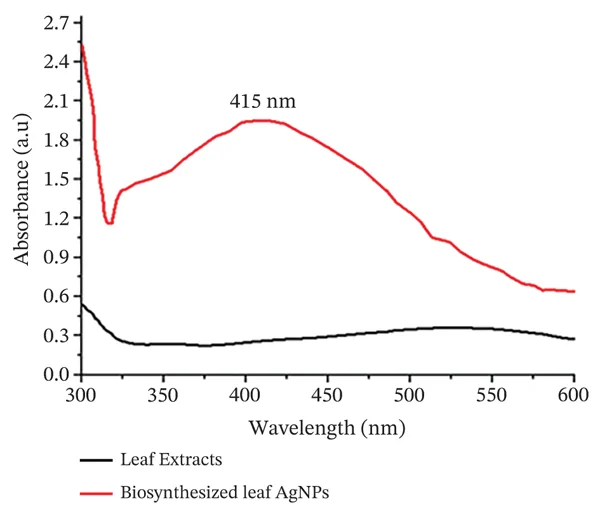
UV‐Vis absorption spectra of leaf extracts and AgNPs synthesized from *Bougainvillea spectabilis* leaves.

The sharpness and symmetry of the SPR at 415 nm reflect nanoparticle stabilization and effectiveness of AgNPs [23]. This is an important observation because the optical properties of metallic AgNPs are highly sensitive to their size, shape and morphology [18]. The position of the absorption peak agrees well with those of other studies carried out on green‐synthesized AgNPs using plant extracts, which usually range between 400 and 450 nm, thus confirming the successful reduction of Ag^+^ to their zerovalent metallic form by the leaf extract of *B*. *spectabilis* [24, 25]. Moreover, the high stability of AgNPs from the capping action of *B*. *spectabilis* extracts; the proteins and phenol compounds in the extract formed a dense protective layer around silver particles. This nature of the capping process introduces steric obstruction and electrostatic repulsion among particles; therefore, it prevents long‐term agglomeration without necessarily requiring chemical stabilizers.

### 3.2. FTIR Analysis and Functional Group Identification

FTIR spectroscopy was used to detect the functional groups in the *B*. *spectabilis* leaf extract potentially responsible for the reduction and capping of AgNPs. Figure 4 shows a comparison of the FTIR spectra of the leaf extract, AgNO_3_ precursor, and AgNPs.

**Figure 4:**
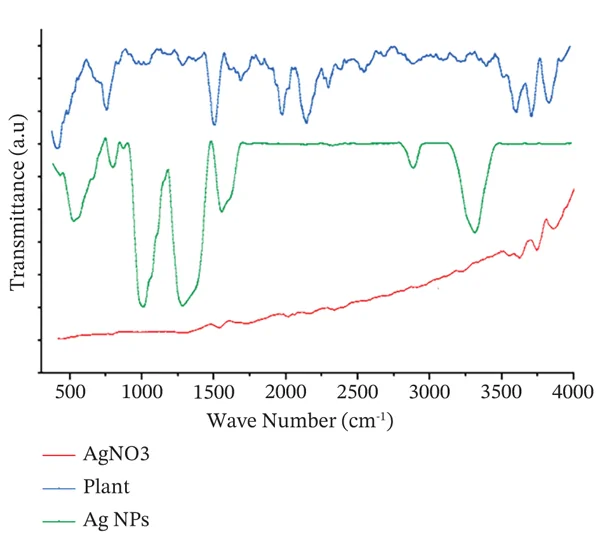
The FTIR of the *Bougainvillea spectabilis* leaf extract, AgNO_3_ precursor, and biosynthesized AgNPs.

The absorption spectra of amide, ketone, or quinone groups were observed, with a peak at ∼3420 cm^−1^ referring to the N–H or O–H bond stretching of alcohols, amines, and phenols. A peak at ∼1640 cm^−1^ reveals to the C=O bond stretching of amides, ketones, or quinones. Furthermore, a peak at ∼1380 cm^−1^ refers to the C–N bond stretching in aromatic amines or C–H bond bending in alkanes, whereas the peak at ∼1050 cm^−1^ corresponding to the C–O bond stretching of alcohol and carboxylic acids.

In the spectrum of the biosynthesized AgNPs, a marked vibrational frequency shift accompanied by a decrease in peak intensity was observed in several peaks. For instance, the broad peak corresponding to O–H/N–H at ∼3420 cm^−1^ in the extract shifted to ∼3400 cm^−1^ in the AgNPs. Also, the peak relating to C=O at ∼1640 cm^−1^ was slightly shifted with decreased intensity. These observations suggest that the amine, hydroxyl, and carbonyl functional groups in the phytoconstituent compounds (flavonoids and terpenoids) participate in reducing the Ag^+^ to the zerovalent Ag^0^ state and surface binding. These functional groups are likely to act like stabilizing agents and capping agents [22]. The spectral shift observed indicates the presence in the spectrum of a stable organic layer formed on silver to prevent agglomeration, which plays a vital role in maintaining colloidal stability [26].

### 3.3. Analysis of XRD

The structure properties of the biosynthesized AgNPs were examined by using XRD as shown in Figure 5. The sharp, high intensity diffraction peaks display the high crystallinity of the nanoparticles. Obviously, the characteristic Bragg reflections detected at 2*θ* values about 27.8°, 32.2°, 46.2°,54.8°, and 76.7° index perfectly to a cubic silver chloride (AgCl) phase (JCPDS No. 31‐1238). Simultaneously, at 38.1°, 44.3°, 64.4°, and 77.5° match to the (111), (200), (220), and (311) lattice planes a face‐centered cubic (FCC) metallic silver lattice (JCPDS No. 04‐0783). This dual‐phase coexistence the formation of a hybrid Ag/AgCl. The AgCl phase residue when a chloride ion (Cl^−^) inside the plant extract reacts with free Ag^+^, whereas residual ions undergo bioreaction to Ag^0^. This dual‐phase interface creates a Schottky barrier that controls charge recombination, which increases chemical reaction [27].

**Figure 5:**
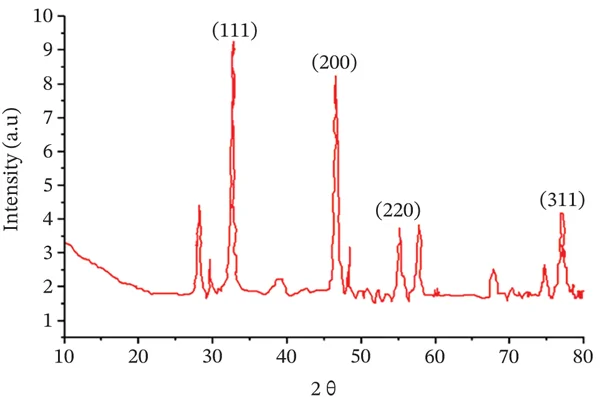
XRD analysis of AgNPs.

In addition, minor peaks were observed at 2*θ* values of 39.1°, 54.5°, and 67.8°. These peaks indicate a slight silver oxide phase of the nanoparticles, probably caused by oxidation during exposure to air or the preparation process [28]. In this work, the average crystallite size of the synthesized AgNPs was calculated to average between 30 and 50 nm using Scherrer′s equation (Equation (1)).

It is necessary to differentiate between the crystalline dimensions and particle sizes; the Scherrer equation estimates the size of coherent crystalline domains, whereas the morphological particle size observed via SEM is typically larger due to the nature of nanoparticles aggregation. This finding confirms the successful synthesis of nanoscale silver particles, with average crystallite dimensions closely aligning with previously reported literature [29, 30].

### 3.4. Analysis of SEM Images

SEM analyses have confirmed the formation of well‐defined AgNPs. Figure 6 shows the SEM image representing the biosynthesized AgNPs with different magnifications. The images reveal that the nanoparticles have a spherical morphology, with an estimated particle diameter extending between 50 and 200 nm. This spherical morphology is usually prevalent in plant‐mediated synthesis and is influenced by the quantity and type of capping agents present in the extract. [31].

**Figure 6:**
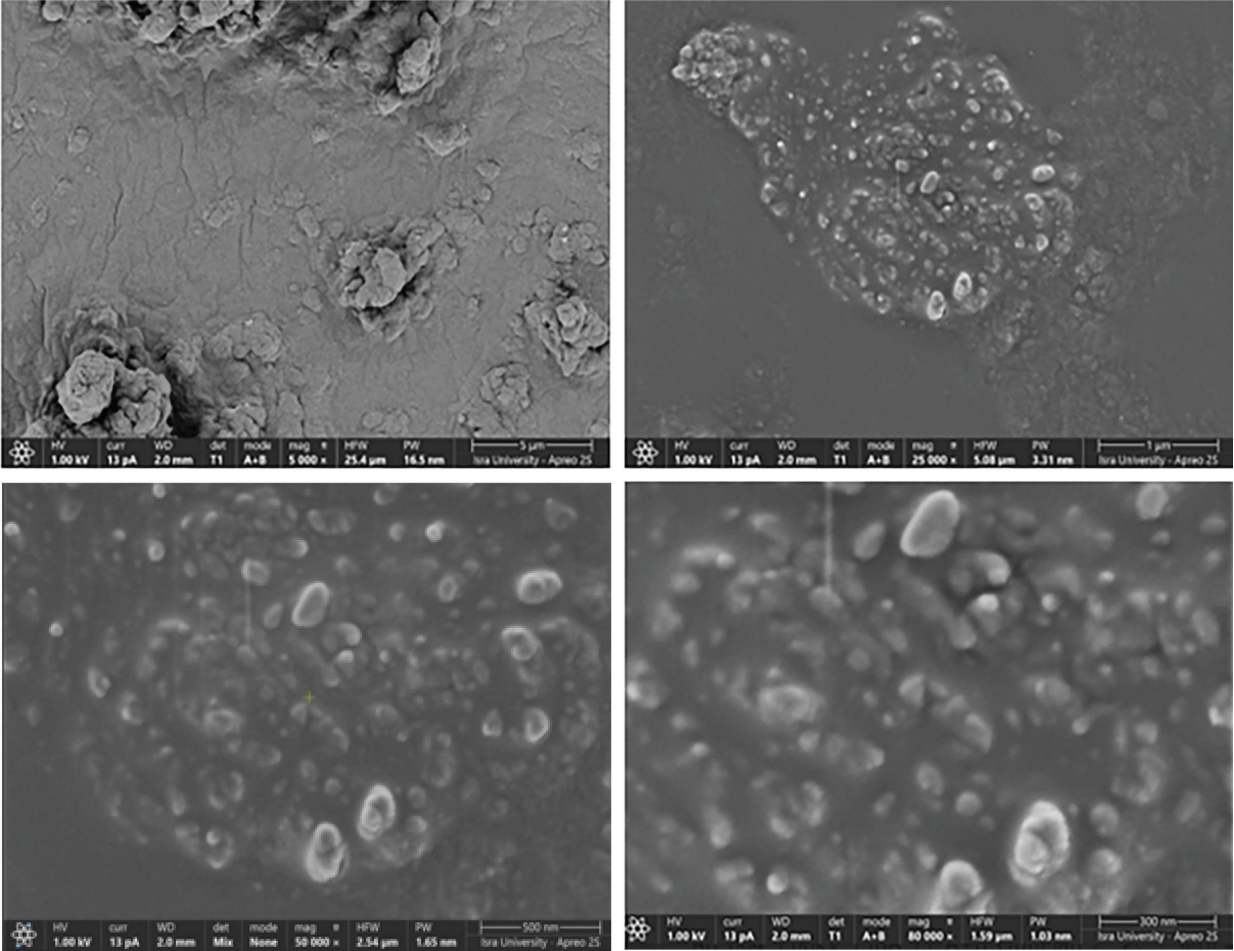
SEM images of synthesized AgNPs with different resolutions showing AgNPs shape and size distribution.

The large particles dimensions observed via SEM compared with the crystallite size calculated from the Debye–Scherrer equation reflect a clear distinction between particle size and crystallite domains. This size variation is primarily attributed to the polycrystalline nature of biogenic AgNPs, where a single nanoparticle comprises multiple smaller crystallites alongside minor agglomeration that occurs during the sample drying process. Furthermore, the codeposition of distinct AgCl and Ag^0^ phases can lead to a larger particle size, although some degree of clustering and agglomeration are visible, which is characteristic of green‐synthesized nanoparticles owing to the evaporation of water and the interaction of organic capping matrices during drying; the micrographs confirm the positive creation of nanoscale structures. The smooth and uniform surfaces of the nanoparticles, with the phytochemical capping layer confirmed through the FTIR analysis, can be highly advantageous for applications requiring a large surface area to be exposed to reactants or pathogens, such as antimicrobial treatment [32, 33].

### 3.5. Evaluation of Antibacterial Activity

The antibacterial possibility of the green‐synthesized AgNPs was tested against the positive *S*. *aureus* and the negative *P*. *aeruginosa* bacteria. The MIC values were determined using the broth microdilution method and presented in Table 1.

**Table 1:** The MIC results of bougainvillea‐synthesized AgNPs and leaf extract against *Staphylococcus aureus* and *Pseudomonas aeruginosa*.

Bacterium	MIC (*μ*g/mL) of AgNPs	MIC (*μ*g/mL) of leaf extract	MIC (*μ*g/mL) of AgNO_3_
*Staphylococcus aureus* (gram‐positive)	6.25	> 200	3.12
*Pseudomonas aeruginosa* (gram‐negative)	12.5	> 200	6.25

The data showed that the synthesized AgNPs have pronounced antimicrobial action against both bacteria with MIC of 6.25 *μ*g/mL against *S*. *aureus* and 12.5 *μ*g/mL against *P*. *aeruginosa*. Additionally, the AgNO_3_ control displayed MIC values of 3.12 and 6.25 *μ*g/mL against *S*. *aureus* and *P*. *aeruginosa*, respectively, representing that although ionic silver is highly toxic, the biogenic AgNPs show comparable and controlled efficacy. This proves that the improved antimicrobial property is mainly because of the synthesized AgNPs, as confirmed in similar analyses conducted in other studies [34].

The synthesized AgNPs displayed greater antibacterial efficacy against the gram‐positive bacteria *S*. *aureus* (indicated by the lower MIC) than against gram‐negative bacteria *P*. *aeruginosa*. This is a well‐observed fact in nanobiology, mostly due to the differences in the structure of the cell walls of negative and positive bacteria [35], gram‐negative bacteria have a specialized outer membrane rich in lipopolysaccharides. This provides a strong impediment to the entry of nano‐cures like AgNPs. The cell walls of gram‐positive bacteria contain mainly a thick layer of peptidoglycan [36].

The mechanism of antibacterial effect of AgNPs is a synergistic effect that involves three main mechanisms. First, the small size and positive charges (due to the release of Ag^+^ ions and their interaction with the negatively charged surface of the bacteria) enable AgNPs to interact with the negatively charged bacterial membranes, thereby causing structural damage also increasing permeability of membrane [37–39]. Secondly, the interaction between the AgNPs and the bacteria causes the production of reactive oxygen species, such as hydroxyl radicals that can cause oxidation to cellular components such as proteins, lipids, and DNA [40]. Finally, the released ions of silver can easily enter the cell and bond with the sulfur‐containing proteins and the DNA with high phosphorus content to inhibit cellular activities such as DNA replication and enzyme action [41, 42]. The low MICs obtained in the current work (6.25 and 12.5 *μ*g/mL) are comparable or even superior to those obtained with AgNPs synthesized employing other plant extracts, highlighting the potential of the bougainvillea‐mediated method for developing antimicrobial agents [43–45].

## 4. Conclusion

In this study, we successfully established the eco‐friendly biosynthesis of AgNPs using the reducing and stabilizing potential of *B*. *spectabilis* leaf extract. The results showed the production of spherical‐shaped AgNPs with a peak at 415 nm. The FTIR results suggest that the phytochemicals (such as hydroxyl and carbonyl functional groups) have potentially performed a significant role in the biosynthesis of AgNPs. Notably, the obtained AgNPs showed strong antimicrobial efficacy against *S*. *aureus* (MIC: 6.25 *μ*g/mL) and *P*. *aeruginosa* (12.5 *μ*g/mL) bacteria. These findings show the sustainability of this green synthesis for producing high‐quality nanomaterial for potential applications in the health and environmental fields.

## Funding

No funding was received for this manuscript.

## Conflicts of Interest

The authors declare no conflicts of interest.

## Data Availability

The data that support the findings of this study are available from the corresponding author upon reasonable request.
